# HLA Desensitization in Solid Organ Transplantation: Anti-CD38 to Across the Immunological Barriers

**DOI:** 10.3389/fimmu.2021.688301

**Published:** 2021-05-20

**Authors:** Nizar Joher, Marie Matignon, Philippe Grimbert

**Affiliations:** ^1^ Assistance Publique-Hôpitaux de Paris AP-HP, Hôpital Universitaire Henri Mondor, Service de Néphrologie et Transplantation, Fédération Hospitalo-Universitaire (Innovative Therapy for Immune Disorders), Créteil, France; ^2^ Université Paris Est Créteil UPEC, Institut National de la Santé et de la Recherche Médicale INSERM U955, Institut Mondor de Recherche Biomédicale IMRB, Équipe 21, Créteil, France

**Keywords:** anti-CD38, daratumumab, HLA desensitization, DSA, solid organ transplantation

## Abstract

The presence of anti-human leucocyte antigen (HLA) antibodies in the potential solid organ transplant recipient’s blood is one of the main barriers to access to a transplantation. The HLA sensitization is associated with longer waitlist time, antibody mediated rejection and transplant lost leading to increased recipient’s morbidity and mortality. However, solid organ transplantation across the HLA immunological barriers have been reported in recipients who were highly sensitized to HLA using desensitization protocols. These desensitization regimens are focused on the reduction of circulating HLA antibodies. Despite those strategies improve rates of transplantation, it remains several limitations including persistent high rejection rate and worse long-term outcomes when compare with non-sensitized recipient population. Currently, interest is growing in the development of new desensitization approaches which, beyond targeting antibodies, would be based on the modulation of alloimmune pathways. Plasma cells appears as an interesting target given their critical role in antibody production. In the last decade, CD38-targeting immunotherapies, such as daratumumab, have been recognized as a key component in the treatment of myeloma by inducing an important plasma cell depletion. This review focuses on an emerging concept based on targeting CD38 to desensitize in the field of transplantation.

## Introduction

### HLA Sensitization and Antibody-Mediated Rejection

Solid organ transplantation (SOT) has become the best therapeutic option for end-stage organ disease but faces two major issues: the limited transplant supply and the poor long-term transplants outcome which have not improved over the past 30 years (1–3). This observation is related to the occurrence of antibody-mediated rejection (ABMR) which remains the death-censored leading cause of transplant loss across all solid organ transplants (3, 4). ABMR is defined on the association of histologic lesions (microvascular inflammation), histologic evidence of alloantibodies–endothelium interaction (c4d staining) and circulating donor-specific antibodies mostly directed against human leucocyte antigens (HLA) (3–10). Following blood transfusion, pregnancy or previous graft failure, candidates for organ transplantation can become sensitized against HLA and produce circulating anti-HLA antibodies (11, 12). In particular, pending on their properties donor-specific anti-HLA antibodies (DSA), are responsible for ABMR leading to allograft dysfunction and graft loss (13–18). Currently, immunomotoring of the transplant candidate’s is routinely performed in order to stratify the immunological risk by determining the presence and specificity of anti-HLA antibodies and potential DSA (11, 16). The highly sensitized patients have longer waitlist times with significant adverse effect on both quality and quantity of life (1, 2). Several strategies are applied to limit the time on the waiting list of highly immunized patients such as prioritization in transplant’s access, promotion of transplantation from living-donor allografts, development of kidney paired donation and desensitization.

### Desensitization and Solid Organ Transplantation’s Outcome

Current desensitization strategies have been developed in kidney transplantation and extended to other solid organ transplantation (17–21). The goal of desensitization regimens in presensitized transplant candidates is twofold including the reduction of anti-HLA level to allow transplantation and the improvement of transplantation outcome through the prevention of ABMR (22). A stepwise approach is commonly used to desensitize including, (i) either high-dose intravenous immunoglobulin (IVIG) or low dose IVIG in association with plasmapheresis to remove antibodies and, (ii) anti-CD20 targeting agent, such as rituximab, to prevent rebound antibodies development by B cell depletion (23–27). Regarding the kidney transplantation field, despite the desensitizing effect, the subsequent transplantation is associated with higher rate of rejection and higher rate of hospital readmission after transplantation (28–30). However, long term outcomes for patient and graft survival have been reported to be similar to that of non-sensitized patients (31). Furthermore, the benefit of desensitization compared to remaining on the transplant waiting list has been evaluated only in few large studies and their results remain controversial (32, 33). Montgomery et al. and Orandi et al. reported a survival benefit at five years after kidney transplantation in 211 and 1025 desensitized patients respectively compared to patients remaining on the waiting list (34, 35). Interestingly, in a study performed on 213 desensitized recipients of living donor transplants, Manook et al. showed that desensitization was not associated with a survival benefit compared to matched sensitized control patients who were waitlisted (36). On the other hand, keeping patients a long time on dialysis represent a considerable financial burden while decreasing the quality and length of life for affected patients (32, 33).Thus, it appear as necessary to develop novel therapeutic approaches in order to prevent ABMR and improve long-term survival of transplanted organs in highly immunized recipient.

### Desensitization Regimens Targeting Plasma Cells

The available therapeutic tools to manage the humoral response appears modestly successful in the context of SOT and alloimmunity. Indeed, antibody rebound due to plasma cells (PC), which do not express CD20, limit the efficacy of the most commonly used strategy combining IGIV, plasmapheresis and B cell depletion by anti-CD20 depleting agent. Targeting PC with new pharmacological tool from autoimmunity and cancer research could allow a better management of the humoral response in desensitization protocols (37). In the germinal center, after the enhancement of alloantigen responses by T follicular helper (Tfh), activated B cells develop into memory-B cells, progress to plasmablasts and ultimately to antibody-producing PC (38, 39). These PC are the long-lived mediators of lasting humoral immunity and persist in medullary niche where they can secrete high-affinity complement-activating DSAs (38, 40). Several emerging strategies aim to deplete PCs compartment in order to prevent ABMR (37, 41). First, Interleukin 6 (IL-6) is a cytokine promoting Tfh and enhancing the progression of B cells to high-affinity antibodies producing PC (42). Tocilizumab, a first-in-class humanized monoclonal antibody (mAb) with specificity for IL-6R, reduce inflammation within the allograft during ABMR in heart and kidney transplantation (43) and induce circulating DSA reduction (44). Clazakizumab is a humanized IgG1 mAb with specificity for IL6 which can also induce circulating DSA reduction (45). Both Tocilizumab and Clazakizumab are pharmacological agents with major interest in the development of desensitization strategies targeting PC (37, 46). On another hand, proteasome inhibitors represent one of the most promising solution to deplete PC in the setting of desensitization, targeting more selectively PCs population. Bortezomib and carfilzomib have been evaluated in desensitization trials, lacking control group, leading to controversial results (47, 48). Both induce significant PCs depletion whereas DSA level did not significantly decrease or rebound occurred rapidly. In fact, targeting PC may lead to rapid germinal center activation by deleting the negative feedback usually provided by PC and rebound humoral immunity and compensation (49). Therefore, dual targeting approach (combining PCs depletion with proteasome inhibitors and costimulation blockade) may silence the germinal center and prevent humoral compensation. This strategy has been recently evaluated using carfilzomib and belatacept as desensitization in highly sensitized non-human primate model with a reduction of bone marrow PC, DSA levels reduction, and prolongation of allograft survival. Most animals experienced ABMR with humoral-response rebound, suggesting desensitization must be maintained after transplantation using ongoing suppression of the B cell response (50, 51). An emerging therapy to induce DSA reduction and to prevent rebound DSA development is the use of antiplasma cell therapies such as anti-CD38, anti-CD19 or bispecific anti-CD3/anti-BCMA (B cell maturation antigen). In this review, we propose to focus on anti-CD38 as a desensitization regimen in SOT.

## CD38-Targeting Strategies

### CD38 and CD38-Targeting Antibodies

The protein CD38 is a type II transmembrane glycoprotein known as a multifunctional molecule. CD38 play dual roles as receptors and ectoenzymes (52). The CD38/CD31 interactions are crucial to leukocyte adhesion and transmigration through the endothelium (53). CD38 is also an enzyme that catalyzes several reactions leading to the regulation of cytoplasmic calcium fluxes and a wide range of others physiological functions such as cellular metabolism (52). CD38, found throughout the immune system especially natural killer and PC, is highly expressed in multiple myeloma cells (54). Altogether, this has triggered the development of several CD38 antibodies to treat multiple myeloma (54–56). Daratumumab (DARZALEX^®^, Janssen), fully human IgG1-kappa, was the first CD38 antibody that was recognized as an emerging therapy against myeloma in the last decade (57). Daratumumab have multiple effects including Fc-dependent immune-effector mechanisms and direct effects. The Fc-dependent immune-effector mechanisms include antibody-dependent cellular cytotoxicity, antibody-dependent cellular phagocytosis, and complement-dependent cytotoxicity (54, 55). Direct effects include induction of apoptosis, as well as inhibition of CD38 ectoenzyme function, which may lead to disruption of the PCs niche. Those Fc-dependent effects and direct effects are associated with deep and sustained CD38^+^ cells depletion, mostly PC and NK cells (54, 55, 58). The ability of daratumumab to efficacy deplete PCs compartment permit to use it as an new agent in therapeutic armamentarium for multiple myeloma (56). Large clinical trials have demonstrated significant improvements in the outcome of patients with relapsed multiple myeloma with use of daratumumab and it has been recently approved in front-line regimens (56–60). Isatuximab (SARCLISA^®^, Sanofi) is a chimeric IgG1-kappa which has stronger direct effects than daratumumab but lower ability to induce Fc-dependent immune-effector mechanisms, while it remains unknown whether these functional differences observed between different CD38 antibodies affect their therapeutic utility (55, 61). Many other strategies targeting CD38 are under development and a selection is listed in **Table 1**. The CD38-targeting antibodies generally represent a safe treatment. Indeed, the most reported toxicity is infusion related reactions which remain successfully controlled by premedication and infusion rate management with low frequency of recurrence during subsequent injections (62). A higher rate of viral infections in patients treated with daratumumab has been reported in some studies leading to a recommended administration of valaciclovir during the administration of anti-CD38 antibodies (62).

**Table 1:** Selection of therapeutical regimens targeting CD38.

Anti-CD38 strategies	Nature and mechanism	Statut	NCT number
**Daratumumab** *Janssen*	Fully human IgG1-kappa anti-CD38 mAb	Approved	X
**Isatuximab** *Sanofi*	Chimeric IgG1-kappa anti-CD38 mAb	Approved	X
**Felzartamab - MOR202** *MorphoSys AG*	Fully human IgG1-lambda anti-CD38 mAb	Ongoing in auto-immune field	NCT04733040 NCT04145440
**Mezagitamab - TAK-079** *Takeda*	Fully human IgG1-lambda anti-CD38 mAb	Ongoing in hemato-oncology	NCT03439280
**CID-103** *CASI Pharmaceuticals*	Fully human IgG1 anti-CD38 mAb	Ongoing in hemato-oncology	NCT04758767
**ISB 1342** *Glennmark Phamaceuticals*	CD3xCD38 bispecific antibody to redirect cytotoxic potential of T cells to CD38^+^ cells	Ongoing in hemato-oncology	NCT03309111
**TAK-169** *Takeda*	Antibody drugs conjugates: anti-CD38 Ab fragment combined to a Shiga-like toxin (payload: ribosome inactivation)	Ongoing in hemato-oncology	NCT04017130
**TAK-573** *Takeda*	Antibody drugs conjugates: humanized IgG4 anti-CD38 mAb combined to interferon *α* (payload: anti-proliferative effects)	Ongoing in hemato-oncology	NCT03215030
**²¹¹At-OKT10-B10** *Fred Hutchinson Cancer Research Center*	Antibody drugs conjugates: anti-CD38 mAb combined to radioactive Astatine ²¹¹At (payload: radiation)	Ongoing in hemato-oncology	NCT04579523 NCT04466475
**STI-6129** *Sorrento Therapeutics*	Antibody drugs conjugates: anti-CD38 mAb combined to Duostatin5 (payload: tubulin inhibition)	Ongoing in hemato-oncology	NCT04316442
**KP1237** *Kleo Pharmaceuticals*	Endogenous-antibodies recruiting molecule targeting CD38 in order to enhance antibody-dependent destruction mechanism	Ongoing in hemato-oncology	NCT04634435
**Anti-CD38 CAR-T Cells** *Sorrento Therapeutics*	Imunne cell therapy based on autologous T cells modified into anti-C38 CAR-T cells	Ongoing in hemato-oncology	NCT03464916

### Immunomodulatory Effects of CD38-Targeting Antibodies

CD38-targeting antibodies have immunomodulatory effects such as improving the host-anti-tumor immune response (63). Krejcik et al. showed that daratumumab monotherapy against myeloma was associated with both CD4+ and CD8+ T cell expansion (64). This increase in T-helper cells and cytotoxic T-cell was associated with functional modification including elevated antiviral and alloreactive functional responses, and significantly greater increases in T-cell clonality as measured by T-cell receptor sequencing (63, 64). These modifications are associated with depletion of CD38^+^ immunosuppressive cells including regulatory T cells, regulatory B cells, and myeloid-derived suppressor cells. It is well known that such regulatory cells inhibit the host-anti-tumor immune response in the context of several malignancies including multiple myeloma (65–67). Altogether, this immunomodulatory activity of CD38 antibodies may be essential to their therapeutic efficacy. Indeed, it has been highlighted in clinical trials showing that expansion of effector T-cells and eradication of immune suppressors cells by daratumumab used against refractory and newly diagnosed multiple myeloma was correlated to a marked improvement in response and progression-free survival (57, 59, 63, 67). It might be hypothesized that these immunomodulatory abilities have important implication for sustained control of the tumor and further deepening of response (63). As a result of these pleiotropic immune modulation, CD38 antibodies also enhance anti-tumor activity of others anti-cancer drugs with several studies highlighting that CD38-targeting antibodies have strong synergistic activity, such as combination to lenalidomide as well as to PD1/PD-L1 inhibitors (56, 68). Besides effect on immune cells, CD38 antibodies may also modulate immunometabolic pathway. Indeed, CD38-targeting agent’s exposure could lead to lower adenosine level in tumoral microenvironment, which is known as immunosuppressive metabolite (69, 70). All these properties enhancing the anti-tumoral response are of major interest in the field of oncology while it could be problematic in immunosuppressive strategies such as autoimmune diseases treatment or desensitization and SOT’s context.

## CD38 Antibodies in Solid Organ Transplantation

### CD38 Antibodies in Non Tumoral Context

In the last decade, several strategies to handle with autoimmune or alloimmune pathologic situations include CD38 antibodies (71–73). Indeed, long-lived plasma cells, which produce pathogenic antibodies, are unresponsive to standard immunosuppression. Besides PC depletion and immunomodulatory effect, CD38 expression on PCs from patients with autoimmune condition (74) and reduction of auto-antibodies in patients exposed to daratumumab (75) support the evaluation of daratumumab in patients with autoantibody-dependent disorders and, in extension, to alloimmune situation such as SOT. Available evidence about CD38 antibodies efficacy in these situations are mostly cases reports of daratumumab use against immune cytopenia. Daratumumab were used to treat warm autoimmune hemolytic anemia post-hematopoietic stem cell transplant (76), refractory cold agglutinin disease (77), Evans syndrome (78) and pure red cell aplasia (79) with improvement in the majority of cases. Regarding other autoimmune disease, the administration of daratumumab in two patients with refractory lupus was recently described exhibiting clinical responses associated with significant depletion of long-lived plasma cells and modulation of effector T-cell responses (80). As regard as autoimmune encephalitis, targeting CD38 was achieved with daratumumab in one case of life-threatening anti-NMDA receptor encephalitis and in one case of refractory anti-CASPR2 encephalitis with improvements of neurological sequelae (81, 82). In the last case, severe septicemia leading to patient death highlight an unmet need of rigorous clinical investigation to determine the efficacy and tolerance of CD38-targeting agent in autoimmune disease.

### CD38 Antibodies and ABMR Treatment

**Figure 1:**
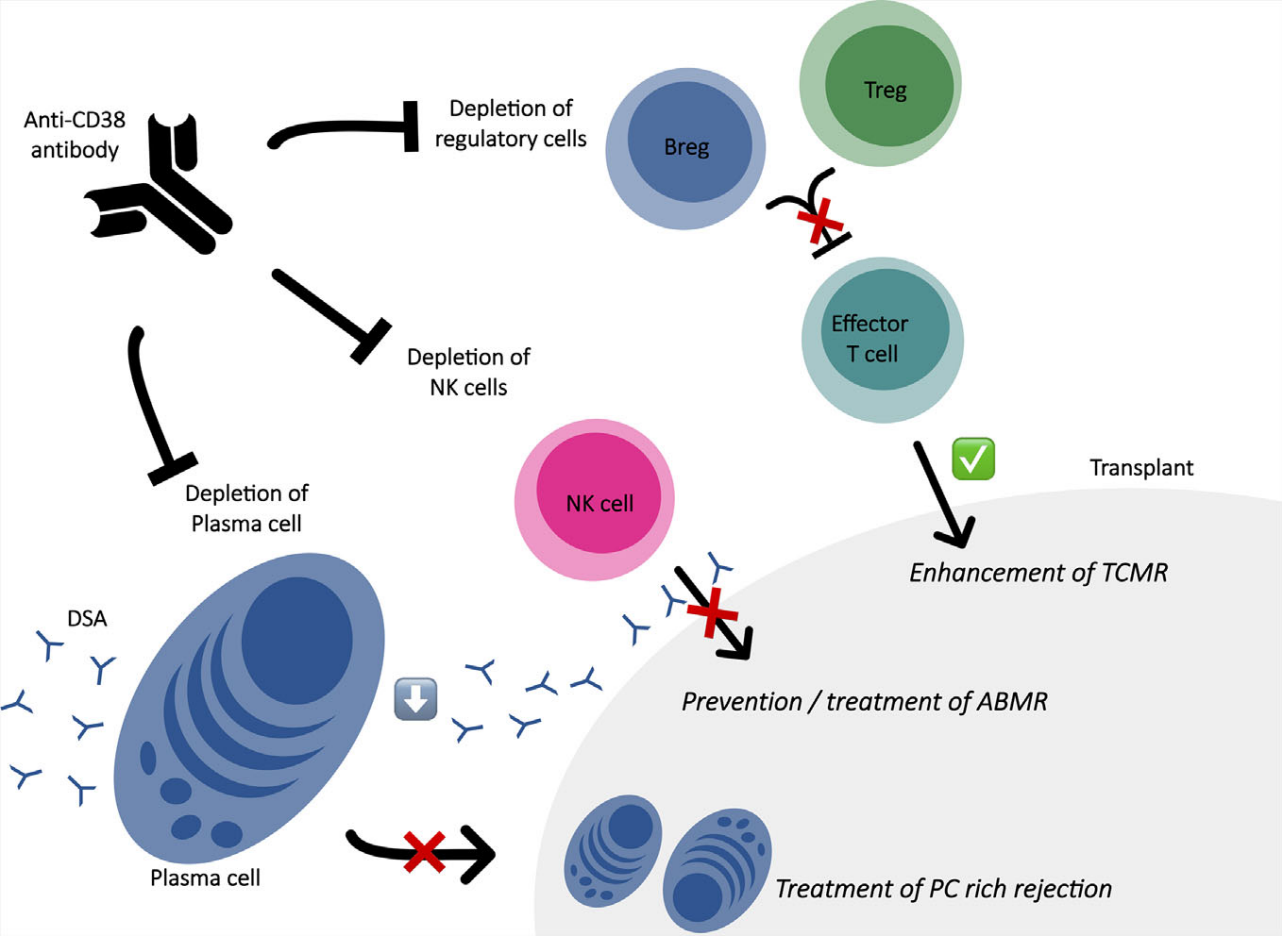
Immune effects of anti-CD38 antibody in the context of solid organ transplantation. ABMR, antibody mediated rejection; Breg, regulatory B cell; DSA, donor specific antibodies; PC, plasma cell; TCMR, T cell mediated rejection; Treg, regulatory T cell.

In antibody-mediated non-neoplastic diseases, alloimmune situation such as SOT represent a field where targeting CD38 is promising as shown in **Figure 1**. As alloantibody-producing PC express CD38 at a higher level than other CD38^+^ hematopoietic cells and CD38 antibodies induce a profound depletion of CD38^+^ PC, CD38 appears as a rational target to handle with harmful alloantibodies such as DSA (83, 84). Currently, only few studies have been published regarding the use of CD38 antibodies for desensitization in patients awaiting transplantation or for treatment of ABMR as shown in **Table 2**. Concerning treatment of ABMR, the first report was in a patient with refractory early active ABMR caused by anti-A isohemagglutinins after kidney transplantation from his ABO-incompatible sister (85). Based on the efficacy of daratumumab in the treatment of pure red cell aplasia following ABO-incompatible hematopoietic stem cell (79) and non-response of several therapies; daratumumab were tested as a rescue solution leading to a significant decrease of the pathogenic isohemagglutinins and resolution of tissue damage in the kidney biopsy. Kwun and colleagues also published a case report of daratumumab as a therapeutic strategy for refractory heart and kidney rejection in a patient who received heart and kidney transplants due to systemic lupus (72). Both transplant biopsy showed T cell–mediated rejection, ABMR and diffuse PC infiltration associated to the presence of several DSA. To face refractory cardiogenic shock and acute kidney failure dependent to dialysis, a compassionate use of daratumumab lead to the resolution of both allograft function, improvement in acute kidney lesions with decreased PCs infiltrate and dramatic decline for the majority of DSA. A recurrent acute PC-rich rejection on kidney biopsy and significant ascension of DSA were successfully managed with daratumumab. Recently, two others cases were reported: one refractory ABMR after a heart transplant successfully treated with daratumumab and one chronic active ABMR in a kidney allograft recipient diagnosed with myeloma exposed to daratumumab (73, 86). In the last one, the exhaustive immuno-monitoring showed that the main mode of action seems to be based on PC depletion, with profound PCs reduction in the bone marrow and peripheral blood and the abrogation of *in vitro* alloantibody production by PC enriched from bone marrow aspirates, leading to significant reduction in DSA levels (73). Another observation is that daratumumab led to depletion of NK cells infiltrating the allograft and circulating NK cells, which is major interest knowing the potential role of NK cells in microvasculature inflammation through engagement of their Fc gamma receptor IIIA with endothelium-bound DSA (87). Interestingly, while follow up biopsy showed resolution of humoral activity, it was observed tubulointerstitial inflammation which prompted steroid treatment. The author highlighted that the molecular signature of this infiltrate was not similar to signature of T-cell mediated rejection leading to question the trigger of this infiltrate not associated with graft dysfunction. Indeed, daratumumab may trigger T-cell alloresponse, even if circulating regulatory T cells were not reduced in the patient’s blood which is not necessarily correlated to the modification of immune cell populations at a tissue level. Moreover, the authors recently reported long term data of this case without evidence of ABMR rebound after daratumumab discontinuation (88). Although it is difficult to decipher the role of a rescue with daratumumab added to a complex antirejection therapy, a drug that specifically deplete PC with a favorable safety profile could represent a step forward in the field.

**Table 2:** CD38 antibody use in solid organ transplantation.

ABMR Treatment								
Réf.	Transplant	Sensitization	IS strategy	Immune event	Treatment	AntiCD38 use	Evolution	Observation
(72)	Heart + Kidney	Immunized: Preformed DSA	- Induction: ATG -Maintenance: + Tacrolimus + MMF + Steroid	*-Delay post-Tx:* 17 months *-Clinical findings:* Cardiogenic shock and acute kidney injury requiring dialysis *-Anti-HLA: de novo* DSA and one preformed DSA *-Histology:* TCMR and ABMR with PC-predominant infiltration in both transplants	Steroid pulses + ATG + Plasmapheresis + IVIG + Rituximab + Eculizumab	Daratumumab: - 16 mg/kg - 8 weekly infusions	*-Clinical:* Heart allograft function returned to baseline + no more need of dialysis *-Anti-HLA:* Dramatic decline of MFI for majority of DSA at 3 months *-Histology:* Significant improvement in acute lesions and the PC infiltrate significantly decreased	-20 weeks after: recurrent acute PC-rich rejection on kidney biopsy -Significant reascension of the MFI of two class 2 DSAs -New series of Daratumumab infusions with kidney allograft function improvement
(73)	Kidney	Immunized: Preformed DSA	- Induction: ? -Maintenance: + Tacrolimus + MMF + Steroid	*-Delay post-Tx:* 13 years *-Clinical findings:* Progressive graft dysfunction and proteinuria in the context of newly diagnosed myeloma *-Anti-HLA:* 1 DSA *-Histology:* chronic active ABMR	None other treatment	Daratumumab: - 16 mg/kg - 8 weekly infusions + 8 fortnightly infusions + 1 monthly infusion thereafter for 9 months	*-Clinical:* Stabilization of renal function and proteinuria *-Anti-HLA:* DSA levels became undetectable after 14 weeks *-Histology:* Abrogation of microvascular inflammation with a decrease of intragraft NK cells densities	-3 months after: subclinical borderline rejection - High-grade tubulitis and mild interstitial infiltrates which were dominated by T-cells -Improvement with high-dose intravenous steroid.
(85)	Kidney	Immunized: ABOi (Anti-A)	- Induction: + Basiliximab + Rituximab -Maintenance: + Tacrolimus + MMF + Steroid	*-Delay post-Tx*: 30 days *-Clinical findings:* acute kidney failure *-Antibodies:* rise in Anti-A titers *-Histology:* ABMR	Steroid pulses + ATG + Immunoadsorption + Eculizumab	Daratumumab: - 16 mg/kg - 6 weekly infusions	*-Clinical:* Recovering of kidney function at baseline *-Anti-A:* Reduction in Anti-A titers leading to discontinuation of immunoadsorption *-Histology:* No lesion	
(86)	Heart	Immunized: History ABMR Preformed DSA	- Induction: ? -Maintenance: + Tacrolimus + MMF + Steroid	*-Delay post-Tx:* 13 years *-Clinical findings:* congestive heart failure *-Anti-HLA:* increase of DSA titers *-Histology:* ABMR	Steroid pulses + Immunoadsorption	Daratumumab: - 16 mg/kg - 8 weekly infusions + 8 fortnightly infusions + 1 monthly infusion thereafter for 9 months	*-Clinical:* Renal and cardiac improvement in 4 weeks *-Anti-HLA:* DSA titers are only slightly reduced -*Histology:* No lesions	
(72)	Preclinical: NHP	Kidney	Daratumumab: -16 mg/kg -4 weekly infusions (8 weeks before Tx)	Plerixafor (anti‐CXCR4): -0.24 mg/kg -same frequency	Significant reduction of DSA levels and prolonged graft survival	None	Induction: anti-CD4 + anti-CD8 Maintenance: Tacrolimus + MMF + Steroid	-Delayed ABMR -DSA rebound -TCMR -Reduction of Breg and Treg -Emergence of activated T cells after kidney transplantation in the desensitization group
(72)	Clinical	Heart	Daratumumab: -16 mg/kg -8 weekly infusions	Plasmapheresis + high-dose IVIG + Rituximab	Significant and persistent reduction of DSA levels and heart transplant access at 6 months	None	NA	Died from surgical complication

ABMR, antibody mediated rejection; ATG, anti-human thymocytes globulins; DSA, donor specific antibodies; IVIG, intravenous immunoglobulins; MMF, mycophenolate mofetil; NHP, nonhuman primate; PC, plasma cells; Ref., reference; TCMR, T cell mediated rejection; Tx, transplantation.

### CD38 Antibodies and Desensitization

The ability of CD38 to desensitize has been evaluated in both preclinical and clinical contexts and published in the same study (72). The preclinical study was based on the use of daratumumab in a non-human primate model which has the most biological similarity to humans for solid organ transplant biology (41, 89). The authors paired donors and recipients for maximal HLA mismatching and practiced, for allosensitization, two serial skin grafts before transplantation with a kidney from paired skin graft donor (72). Daratumumab and plerixafor (anti‐CXCR4), known to induce mobilization of PC from bone marrow to peripheral blood, were given as desensitization therapy with an initiation 8-12 weeks after sensitization and 8 weeks before kidney transplantation. Animals received for induction anti-CD4 and anti-CD8 antibodies and for maintenance immunosuppression tacrolimus, mycophenolate mofetil and a methylprednisolone taper. This desensitization regimen reduced significantly preformed DSA, with more than 50% reduction compared with the pretreatment time point, and prolonged graft survival with a depletion of PC without altering the germinal center response since the Tfh population was not eliminated (72). However, desensitized monkeys showed delayed ABMR associated to DSA rebound and T cell–mediated rejection perhaps due to immune deviation. Indeed, the authors observed a reduction of regulatory B and T cells after desensitization with rapid emergence of activated T cells after kidney transplantation. This observation could be related to immunomodulatory effects of daratumumab but CXCR4 inhibition, due to plerixafor, is also known to limit regulatory compartment and to promote effector cells with a potential role in these cell‐mediated rejection (90). Thus, in transplant recipients following desensitization with daratumumab, it would be interesting to elaborate new strategies than current immunosuppressive regimens in order to manage these DSA rebounds and the risk of T cell–mediated rejection. Concerning the clinical setting, the authors used daratumumab in a heart transplant candidate remaining highly sensitized after multiple courses of plasmapheresis, high-dose IVIG, and rituximab. It was observed a significant and persistent decrease of allosensitization allowing a heart transplantation six months after daratumumab infusion (72). Currently, based on these promising results, daratumumab are under investigation for desensitization in patients awaiting solid-organ transplantation in two clinical trial, one ruled by the nephrology department of Henri Mondor Hospital (Créteil, France) and another one directed by Stanford University [ClinicalTrials.gov, NCT04204980 and NCT04088903 (91, 92)]. Regarding the trial in kidney transplantation, sensitized patients with calculated panel reactive antibodies (cPRA) > 95% awaiting on the French National kidney allograft waiting-list for at least three years are eligible for the study and are randomly assigned to one of the two steps: (step 1) dose-escalation with 4 mg/kg of daratumumab weekly for four weeks, then with 8 mg/kg weekly for four weeks and then 16 mg/kg weekly for four weeks; (step 2) expansion cohort with eight weekly doses of 16 mg/kg. The primary outcomes are defined as: adverse events, intra-patient variation of cPRA and anti-HLA levels. Several other outcomes are also of interest such as percentage of patients engrafted, and intra-patient variation of ABO antibody titers (91).

## Conclusion

Therapeutic improvement is required for both prevention and treatment of humoral alloresponse in solid organ transplantation. CD38 antibodies are a promising solution to profoundly deplete high affinity anti-HLA producing plasma cells. Preclinical and clinical experimental results suggests that daratumumab is a potentially therapeutic strategy to reduce DSA production and prevent and/or treat antibody-mediated rejection. However, CD38-targeting agent induce immune deviation which could be deleterious for solid organ transplants enhancing cellular-mediated rejection. Clinical studies are now needed to clarify the indications and efficacy of these promising therapeutic strategies.

## Author Contributions

NJ, MM, and PG designed the review, collected and interpreted data from literature, and wrote the manuscript. All authors contributed to the article and approved the submitted version.

## Conflict of Interest

The authors declare that the research was conducted in the absence of any commercial or financial relationships that could be construed as a potential conflict of interest.
